# Checkpointinhibitoren in der Tumortherapie

**DOI:** 10.1007/s00103-020-03221-9

**Published:** 2020-10-01

**Authors:** Hilke Zander, Susanne Müller-Egert, Michal Zwiewka, Steffen Groß, Ger van Zandbergen, Jörg Engelbergs

**Affiliations:** Paul-Ehrlich-Institut, Paul-Ehrlich-Str. 51–56, 63225 Langen, Deutschland

**Keywords:** Checkpointinhibitor, Immunonkologie, PD-1/PD-L1, CTLA‑4, Biomarker, Checkpoint inhibitor, Immuno-oncology, PD-1/PD-L1, CTLA‑4, Biomarker

## Abstract

Mit der Entwicklung von Checkpointinhibitoren gelang in den letzten Jahren ein Durchbruch in der Tumortherapie. Checkpointinhibitoren aktivieren die Immunabwehr gegen Tumoren, indem sie die immunhemmende Wirkung spezifischer, als Kontrollpunkte agierender Zelloberflächenproteine, der sogenannten Checkpoints, aufheben. Dieser Artikel gibt einen Überblick über die Wirkweise und den Stand der derzeitigen klinischen Entwicklung zugelassener Checkpointinhibitoren.

Die bisher zugelassenen Checkpointinhibitoren, gegen die Checkpoints CTLA‑4 und PD-1/PD-L1 gerichtete monoklonale Antikörper, werden in verschiedenen Tumorentitäten wie Melanom, Lungen‑, Nieren‑, Urothelkarzinom oder Kopf-Hals-Tumoren sowie dem Hodgkin-Lymphom eingesetzt. Bei einem Teil dieser Patienten mit fortgeschrittenen Tumoren konnte erstmals ein Langzeitüberleben erzielt werden. In Abhängigkeit von der Tumorindikation ist diese charakteristische lange Wirksamkeit jedoch nur bei einem geringen Anteil der behandelten Patienten zu beobachten, was man durch eine Patientenselektion über prädiktive Biomarker und die Entwicklung von Kombinationstherapien zu überwinden versucht. Für manche Indikationen wurde bereits mit der Checkpointinhibitorzulassung eine Einschränkung hinsichtlich des prädiktiven PD-L1-Status vorgeschrieben.

## Abstract (en)

In recent years, a breakthrough in tumor therapy was achieved with the development of checkpoint inhibitors. Checkpoint inhibitors activate the immune defense against tumors by overcoming the inhibitory effect of specific cell surface proteins acting as control points, the so-called checkpoints. This article provides an overview of the mode of action of approved checkpoint inhibitors and the status of current clinical development.

The previously approved checkpoint inhibitors, monoclonal antibodies directed against the checkpoints CTLA‑4 and PD-1/PD-L1, are used in various tumor entities (including lung, kidney, and urothelial carcinoma; head and neck cancer; melanoma; and Hodgkin lymphoma). For the first time, long-term survival has been achieved in some of these patients with advanced tumors. Unfortunately, this efficacy can be observed only in a small proportion of the treated patients, depending on the tumor indication. Improved efficacy is envisioned by patient selection via predictive biomarkers and the development of combination therapies. Mandatory testing of the expression level of the predictive PD-L1 biomarker is already required in some indications to select patients with an enhanced benefit/risk relationship.

## Einleitung

Seit vielen Jahren ist bekannt, dass das humane Immunsystem prinzipiell in der Lage ist, Tumorzellen zu bekämpfen. Das Immunsystem lässt sich in ein evolutionsbiologisch älteres, unspezifisches und in ein jüngeres, spezifisches oder adaptives Immunsystem aufteilen, wobei auch Interaktionen zwischen beiden Komponenten bestehen. Immuntherapeutische Ansätze sind sowohl für das unspezifische als auch für das adaptive Immunsystem seit Langem in klinischer Erforschung. Mit der Entwicklung der sogenannten Checkpointinhibitoren, die die spezifische T‑Zellaktivität (adaptives Immunsystem) beeinflussen, gelang erst der eigentliche Durchbruch in der Immuntherapie. Die Immunforscher Tasuku Honjo und James Allison entdeckten mit den ersten 2 sog. Checkpoints, CTLA‑4 und PD‑1, ein zentrales Regulationsprinzip des Immunsystems und erhielten dafür 2018 den Nobelpreis für Medizin und Physiologie. Im Folgenden wird daher auf die T‑zellregulierenden Checkpointinhibitoren eingegangen.

## Wirkmechanismus des adaptiven Immunsystems

Eiweißstrukturen, die an der Tumorzelloberfläche vorkommen oder von Tumorzellen freigesetzt werden, können von Zellen des Immunsystems als fremd erkannt werden. In dem Fall werden sie Tumorantigene genannt. Um eine solche Struktur als fremd zu erkennen, muss sie dem Immunsystem von sogenannten antigenpräsentierenden Zellen (APC) „vorgestellt“ werden. APCs, insbesondere dendritische Zellen (DC), können diese Tumorantigene aufnehmen und an ihrer Zelloberfläche präsentieren. Durch die Interaktion von dendritischen Zellen mit T‑Helferlymphozyten über den T‑Zellrezeptor (TCR) und das MHC-Klasse-II-Molekül (Major Histocompatibility Complex) werden diese antigenspezifisch stimuliert. Daraufhin vermehren sich T‑Lymphozyten und wandern in die Tumorregion ein (tumorinfiltrierende Lymphozyten, TIL).

**Figure 1:**
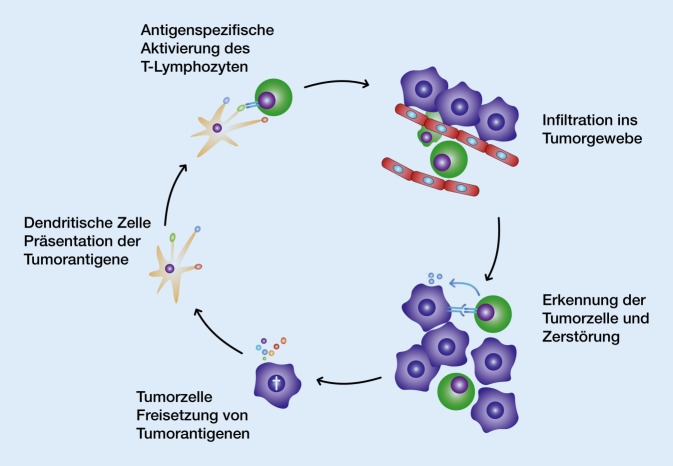

Im Tumorgewebe erkennen die T‑Zellen die auf der Zelloberfläche des Tumors präsentierten Antigene über die Bindung des T‑Zellrezeptors und des MHC-Klasse-I-Moleküls. Durch diese spezifische Bindung wird die zytotoxische T‑Zelle (CTL) aktiviert. Sie kann die Tumorzelle hauptsächlich über die Freisetzung des porenbildenden Proteins Perforin und die Einschleusung von Granzymen in die perforierte Tumorzelle zerstören (Abb. 1 und 2).

**Figure 2:**
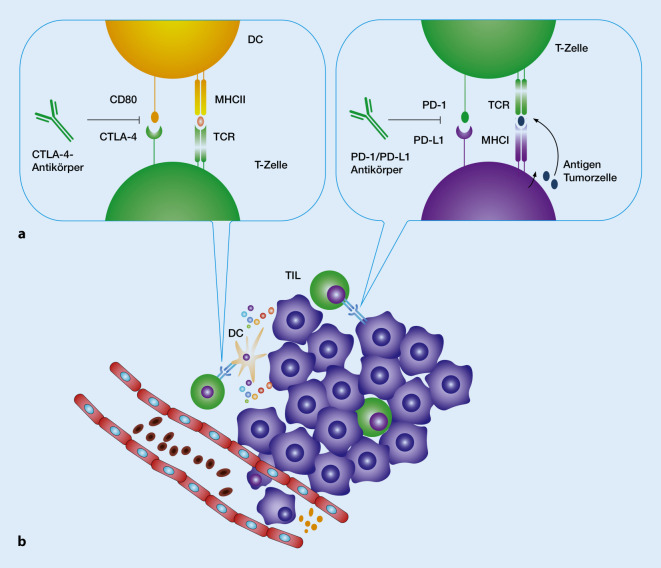

## Regulation einer Immunantwort über Checkpoints

Immunantworten sind durch ein bemerkenswertes gegenseitiges Kontrollsystem über spezifische Kontrollpunkte, sogenannte Checkpoints reguliert [2]. Das natürliche Ziel ist das Eindämmen einer überschießenden Immunantwort, z. B. nach Eliminierung eines Fremdantigens (wie nach dem Abklingen einer viralen Infektion) oder die Vermeidung eines Angriffs auf eigene Körperzellen.

Tumoren nutzen nun diese Checkpoints auf geschickte Weise aus, um eine Immunantwort gegen sich selbst zu verhindern.

Checkpoints regulieren sowohl die Stimulation der T‑Helferzellen durch die antigenpräsentierenden Zellen als auch die Aktivierung der zytotoxischen T‑Zellen (CTL). Die bisher am umfangreichsten untersuchten Checkpoints sind CTLA-4/CD80 oder CD86 und PD-1/PD-L1 oder PD-L2 (CTLA-4: *C*ytotoxic *T*-*L*ymphocyte-*a*ssociated Protein-*4*; PD-1: *P*rogrammed *D*eath *P*rotein-*1*; PD-L1: *P*rogrammed *D*eath *L*igand-*1*). Die Interaktion von CTLA‑4 auf der T‑Zelle mit CD80 (oder CD86) auf der antigenpräsentierenden Zelle hemmt die Stimulierung der T‑Zelle durch die antigenpräsentierende Zelle (DC). Die Interaktion von PD-L1 auf der Tumorzelle mit PD‑1 auf der tumorinfiltrierenden T‑Zelle führt zu einer Hemmung der zytotoxischen Aktivität der T‑Zelle (Abb. 2).

## Wirkmechanismus Checkpointinhibitoren

Mit der Entwicklung eines monoklonalen Antikörpers gegen CTLA‑4 und der Zulassung von Ipilimumab in 2011 gelang ein Durchbruch in der Tumortherapie. Es konnte erstmals gezeigt werden, dass Eingriffe in das immunologische Kontrollsystem therapeutisch nutzbar sind und selbst bei fortgeschrittenen Tumorerkrankungen zu einem Langzeitüberleben führen können.

Der monoklonale Antikörper Ipilimumab bindet an CTLA‑4 auf der T‑Zelle. Dadurch wird die Bindung des CTLA‑4 an CD80 oder CD86 auf der antigenpräsentierenden Zelle verhindert und somit die hemmende Wirkung dieser Interaktion aufgehoben. T‑Zellen können dadurch wieder vermehrt durch antigenpräsentierende Zellen stimuliert werden und gegen den Tumor wirksam werden.

Durch die Entwicklung von monoklonalen Antikörpern gegen PD‑1 oder PD-L1 wurde der Einsatz von immuntherapeutischen Ansätzen in der Onkologie weiter entscheidend vorangebracht. In Europa erhielten Nivolumab und kurz darauf Pembrolizumab in 2015 die Zulassung als PD-1-Inhibitoren, Atezolizumab wurde 2017 als erster PD-L1-Inhibitor zugelassen.

Die genannten Antikörper gegen PD‑1 oder PD-L1 verhindern die Bindung zwischen PD‑1 und PD-L1 (oder PD-L2) und stellen die gegen den Tumor gerichtete zytotoxische T‑Zellantwort wieder her (Abb. 2).

## Klinische Ergebnisse mit Checkpointinhibitoren

**Figure 3:**
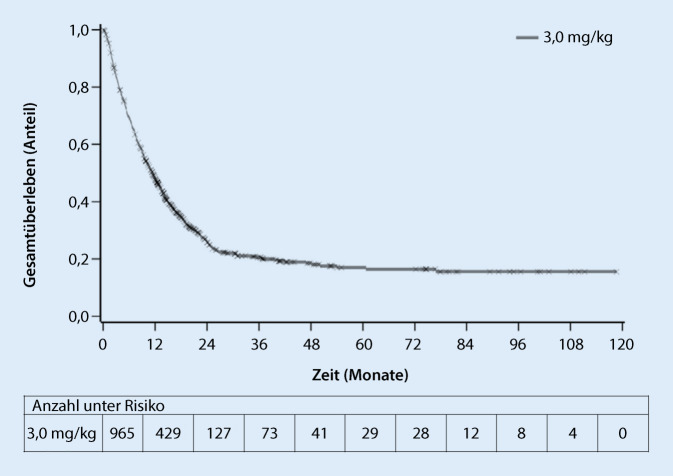

Durch den Einsatz der Checkpointinhibitoren konnte bei Patienten mit fortgeschrittenen soliden Tumoren erstmals ein Langzeitüberleben in einem signifikanten Anteil der Patienten erzielt werden. Die Abb. 3 demonstriert exemplarisch den Langzeitüberlebensvorteil einer Behandlung mit Ipilimumab in einer Metaanalyse aus klinischen Studien bei behandlungsnaiven und vorbehandelten Patienten mit fortgeschrittenem Melanom. Die Kaplan-Meier-Kurve des Gesamtüberlebens zeigt ein Plateau ab etwa dem Jahr 3 (Überlebensrate ca. 21 %), das sich bei einigen Patienten bis in das Jahr 10 erstreckt (Abb. 3; Auszug aus der SmPC Yervoy [3]).

Dieses außergewöhnlich lange Ansprechen der Patienten im palliativen Therapiesetting, das durch den Einsatz von Chemotherapeutika bis dato nie erreicht wurde, weckte große Hoffnungen hinsichtlich der potenziellen Möglichkeiten einer Immuntherapie.

Die Überlegenheit der Checkpointinhibitoren hinsichtlich eines lang anhaltenden Ansprechens konnte zwischenzeitlich in verschiedenen soliden Tumorindikationen sowie bei Patienten mit Hodgkin-Lymphom gezeigt werden. Tab. 1 gibt einen Überblick über die derzeit in Europa zugelassenen Indikationen von Checkpointinhibitoren (SmPCs [3–9]; Stand April 2020).

**Table 1:** 

Target	Wirkstoff/Handelsname (Zulassungsinhaber)	Zugelassene Indikation als Monotherapie	Zugelassene Indikation als Kombinationstherapie
CTLA‑4	Ipilimumab/Yervoy® (BMS)	Melanom	+ Nivolumab in Melanom; Nierenzellkarzinom (1L)
PD‑1	Nivolumab/Opdivo® (BMS)	Melanom	+ Ipilimumab in Melanom; Nierenzellkarzinom (1L)
		Nichtkleinzelliges Lungenkarzinom (2L+)	
		Nierenzellkarzinom (2L+)	
		Hodgkin-Lymphom (3L+)	
		Plattenepithelkarzinom des Kopf-Hals-Bereichs (2L+)	
		Urothelkarzinom (2L+)	
	Pembrolizumab/Keytruda® (MSD)	Melanom	+ Chemotherapie in nichtkleinzelligem Lungenkarzinom (1L)
		Nichtkleinzelliges Lungenkarzinom 1L für PD-L1 TPS ≥50 % 2L für PD-L1 TPS ≥1 %	
		Plattenepithelkarzinom der Kopf-Hals-Region 1L für PD-L1 CPS ≥ 1 2L+: PD-L1 TPS ≥50 %	+ Chemotherapie in Plattenepithelkarzinom der Kopf-Hals-Region (1L) für PD-L1 CPS ≥ 1
		Hodgkin-Lymphom (3L+)	
		Urothelkarzinom 2L+ für alle 1L, nicht für Therapie mit Cisplatin geeignet, für PD-L1 CPS ≥10	+ Axitinib in Nierenzellkarzinom (1L)
	Cemiplimab/Libtayo® (Sanofi)	Kutanes Plattenepithelkarzinom	–
PD-L1	Atezolizumab/Tecentriq® (Roche)	Nichtkleinzelliges Lungenkarzinom (2L+)	+ Chemotherapie (± Bevacizumab) in nichtkleinzelligem Lungenkarzinom mit nichtplattenepithelialer Histologie (1L) + Chemotherapie in kleinzelligem Lungenkarzinom + Chemotherapie in dreifach negativem Mammakarzinom (1L) für PD-L1 ≥ 1 % (auf IC)
		Urothelkarzinom 2L+ für alle 1L, nicht für Therapie mit Cisplatin geeignet, für PD-L1 ≥ 5 % (auf IC)	
	Avelumab/Bavencio® (Merck)	Merkelzellkarzinom	+ Axitinib in Nierenzellkarzinom (1L)
	Durvalumab/Imfinzi® (AstraZeneca)	Nichtkleinzelliges Lungenkarzinom bei stabiler Erkrankung nach platinbasierter Radiochemotherapie PD-L1 ≥ 1 % (auf TC)	–

*1L* Erstlinientherapie; *2L+* zumindest Zweitlinientherapie (nach Versagen einer Erstlinientherapie); PD-L1-Zulassung beschränkt auf PD-L1-exprimierende Tumoren; *TPS* Tumor Proportion Score (d. h. prozentualer Anteil PD-L1-positiver Tumorzellen einer Gewebeprobe); *CPS* kombinierter positiver Score (d. h. PD-L1-Expression auf Tumorzellen oder tumorinfiltrierenden Immunzellen); *IC* tumorinfiltrierende Immunzelle; *TC* Tumorzelle

## Probleme bei der Behandlung mit Checkpointinhibitoren

**Figure 4:**
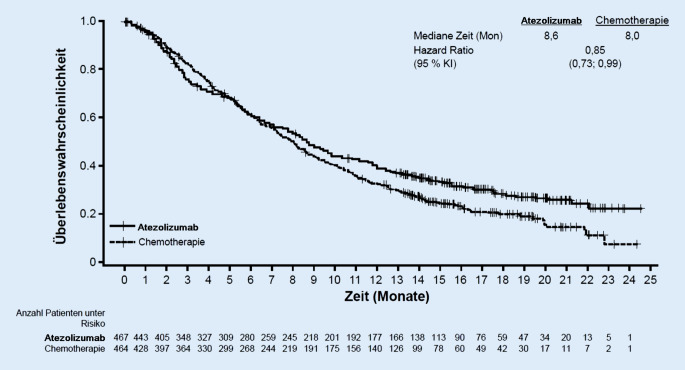

Trotz der für die Immuntherapie charakteristischen langen Wirksamkeit spricht leider nur ein Teil der behandelten Patienten an. In klinischen Studien wurde bei soliden Tumoren eine Ansprechrate von 13 % (bei Zweitlinien- oder späterer Behandlung [2L+]) bei Plattenepithelkarzinom des Kopf-Hals-Bereichs oder Urothelkarzinom, bis zu maximal 40 % (bei Erstlinientherapie [1L] Melanom) oder 45 % (bei 1L nichtkleinzelligem Lungenkarzinom mit einer Tumor-PD-L1-Expression von ≥50 %) durch eine Monotherapie eines Checkpointinhibitors erreicht. Die Beurteilung eines Vorteils der Immuntherapie hängt entscheidend von der Wirksamkeit der in der jeweiligen Indikation verfügbaren Standardtherapie ab. Typischerweise ist eine Chemotherapie durch eine initial schnelle Wirksamkeit bei jedoch zumeist vergleichsweise kurzer Dauer des Ansprechens charakterisiert. Durch diese unterschiedlichen Wirkweisen kann eine Immuntherapie für einzelne Patienten gegenüber einer Standardtherapie nachteilig sein. In Studien drückt sich das durch sogenannte kreuzende Überlebenskurven aus, die einen Überlebensnachteil für die Immuntherapie gegenüber der Chemotherapie in den ersten Monaten des Beobachtungszeitraums zeigen. Dieser Kurvenverlauf lässt sich beispielweise bei dem Einsatz einer Checkpointinhibitormonotherapie bei Patienten mit vorbehandeltem, lokal fortgeschrittenem oder metastasiertem Urothelkarzinom veranschaulichen (Abb. 4; [6]).

Leider lässt sich ein Therapieerfolg für den einzelnen Patienten bisher noch nicht ausreichend vorhersagen. Im Rahmen der klinischen Studien wird nach Biomarkern gesucht, die eine solche Vorhersage ermöglichen würden (siehe unten). Generell scheinen schlechte prognostische Faktoren wie hohe Tumorlast, rasch progredientes Tumorwachstum sowie schlechter Allgemeinzustand eher ungünstig hinsichtlich des Ansprechens einer Immuntherapie zu sein. Zudem geht man davon aus, dass Checkpointinhibitoren ausreichend Zeit brauchen, um wirksam werden zu können.

Durch das Einwandern von Immunzellen in die Tumorregion im Rahmen einer Immuntherapie kann es zu einer anfänglichen Vergrößerung des Tumorvolumens in den bildgebenden Verfahren kommen, die jedoch nicht Ausdruck einer echten Tumorprogression ist. Um zu vermeiden, dass dieses Phänomen einer sog. Pseudoprogression zum vorzeitigen Therapieabbruch führt, wurden spezifische Kriterien für die Bewertung des Ansprechens einer immuntherapeutischen Behandlung bei soliden Tumoren, sogenannte Immune-related-RECIST-Kriterien (iRECIST) entwickelt, die bei klinischen Hinweisen auf einen Nutzen der Therapie deren initiale Fortführung trotz Tumorvergrößerung erlauben.

Ein weiteres Problem bei der Behandlung stellen die spezifischen therapieassoziierten Nebenwirkungen dar, die sich von dem Toxizitätsprofil einer Chemotherapie unterscheiden. Durch die Verschiebung der Balance zwischen aktivierenden und hemmenden Signalen des Immunsystems können immunologisch bedingte unerwünschte Nebenwirkungen (Immune-related Adverse Events, irAEs) auftreten. Prinzipiell können alle Organe betroffen sein und klassische Autoimmunerkrankungen widerspiegeln, wie z. B. Thyreoiditis, Pneumonitis, Hepatitis oder Kolitis. Die Symptome können verzögert, selbst nach Absetzen der Therapie auftreten und im klinischen Alltag kann eine Abgrenzung gegenüber einer Progression der Grunderkrankung Schwierigkeiten bereiten [10, 11].

## PD-L1 als prädiktiver Biomarker

In einzelnen Indikationen hat sich die Expressionsrate von PD-L1 im Tumorgewebe als Indikator für das Ansprechen einer PD-1/PD-L1-Therapie gezeigt (d. h. PD-L1 als prädiktiver Biomarker). In den meisten Indikationen ist eine PD-L1-Expression auf Tumor- und/oder Immunzellen darüber hinaus aber auch therapieunabhängig mit dem Gesamtüberleben assoziiert (d. h. PD-L1 als prognostischer Biomarker).

**Figure 5:**
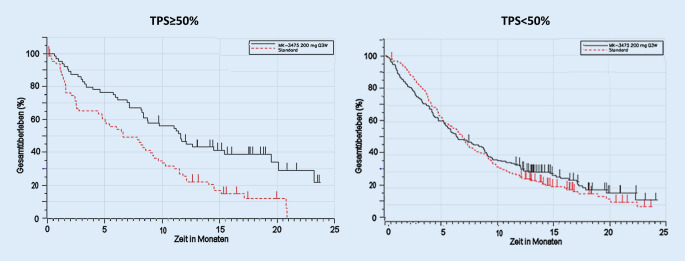

Exemplarisch lässt sich das an dem Beispiel der KEYNOTE-040-Studie [12] veranschaulichen. Patienten mit rezidivierendem oder metastasierendem Plattenepithelkarzinom der Kopf-Hals-Region (HNSCC) nach Progression unter einer vorherigen platinbasierten Therapie wurden entweder mit Pembrolizumab oder einer Standardtherapie nach Wahl des Prüfarztes (Methotrexat, Docetaxel oder Cetuximab) behandelt. Patienten mit einer Tumor-PD-L1-Expression von ≥50 % hatten deutlich größere Überlebensvorteile von einer Behandlung mit Pembrolizumab als die Patienten, in deren Tumoren sich eine niedrigere PD-L1-Expression nachweisen ließ. Für Patienten mit PD-L1 TPS ≥50 % zeigte sich sowohl ein besseres Ansprechen im Pembrolizumabbehandlungsarm als auch ein schlechteres Überleben im Chemotherapiearm im Vergleich zu Patienten mit PD-L1 TPS <50 % (Abb. 5).

Dieses unterschiedliche Ansprechen bei PD-L1 hoch und niedrig exprimierenden Tumoren hat in dem oben genannten Beispiel (wie auch in anderen Indikationen, Tab. 1) zu einer Einschränkung der Zulassung für Patienten mit PD-L1 hoch exprimierenden Tumoren geführt.

In der klinischen Praxis bedeutet das, dass für Indikationen, für die eine Einschränkung hinsichtlich des PD-L1-Status besteht, die PD-L1-Expressionsstärke von Tumorproben vor dem Einsatz von Checkpointinhibitoren mit einem dafür geeigneten, analytisch validierten Test bestimmt werden muss.

## Behandlung mit personalisierten (stratifizierten) Checkpointinhibitoren: Herausforderungen in der klinischen Praxis

In den pivotalen klinischen Studien für Checkpointinhibitoren, die nur selektiv für solche Patienten zugelassen wurden, deren Tumore eine spezifische PD-L1-Expression aufwiesen, wurden checkpointinhibitor- und indikationsspezifisch signifikant unterschiedliche prädiktive Schwellenwerte (Cut-offs) und Auswertungsalgorithmen (Scoring) festgelegt, um eine niedrige oder hohe PD-L1-Expression zu definieren (Tab. 1). Zudem wurden auch unterschiedliche, analytisch spezifisch validierte immunhistochemische (IHC‑)Tests verwendet. Bei Pembrolizumab wurde beispielsweise der PD-L1 IHC-22C3-pharmDx-Test eingesetzt (SmPC Pembrolizumab [4]), der PD-L1 sowohl auf Tumorzellen (TC) als auch auf tumorinfiltrierenden Immunzellen (IC) messen kann. Bei der Kombination von Nivolumab und Ipilimumab wurde hingegen der PD-L1-IHC-28-8-PharmDx verwendet (SmPC Nivolumab [5]), der PD-L1 nur auf TC erfassen kann. Ein Wechsel des PD-L1-IHC-Testsystems während der klinischen Checkpointinhibitorentwicklung (z. B. vom Prototyp zum pivotalen Test) erfordert aufgrund der spezifischen Cut-offs und Scoringalgorithmen (TC, IC, TC+IC) umfangreiche analytische Konkordanzstudien. PD-L1-Tests wurden auch zusammen mit dem jeweiligen Checkpointinhibitor als kommerzielle CE-zertifizierte In-vitro-Diagnostika (IVD) entwickelt und stehen für die klinische Routinetestung zur Verfügung.

Fazit: Das jeweilige Checkpointinhibitorarzneimittel und der dazugehörige spezifische prädiktive PD-L1-biomarkerbasierte IHC-Test bilden eine Art untrennbares „Tandem“. Dieses stellt eine besondere Herausforderung an die klinische Routinetestung dar, da die verfügbaren, spezifisch validierten PD-L1-IHC-Tests aufgrund der jeweiligen PD-L1-Detektion auf unterschiedlichen Zellen (TC, IC, TC+IC) und den entsprechenden checkpointinhibitorspezifischen Cut-offs und Scoringalgorithmen nicht einfach austauschbar sind.

## Ausblick: neue prädiktive Biomarker

Obgleich bisher die PD-L1-Expressionsstärke auf Proteinebene als prädiktiver Biomarker bei zugelassenen Checkpointinhibitoren evaluiert wurde, ist PD-L1 kein optimaler Biomarker, da die PD-L1-Expression dynamisch, heterogen und vom Zelltyp bzw. der Indikation abhängig ist und weiterhin auf unterschiedlichen IHC-Testsystemen und Cut-offs/Scoringalgorithmen beruht.

In jüngster Zeit wurden daher neue, PD-L1-unabhängige Biomarker in zahlreichen klinischen Studien hinsichtlich eines verbesserten prädiktiven Potenzials evaluiert, wie beispielsweise die Tumormutationslast (Tumor Mutational Burden, TMB) analysiert mittels Next Generation Sequencing. Hochmutierte Tumoren (d. h. mit hohem TMB) generieren mit höherer Wahrscheinlichkeit Neoantigene und können dadurch eine erhöhte Immunogenität, verbunden mit einer gesteigerten T‑Zellreaktivität und antitumoralen Antwort, aufweisen. Mehrere Studien konnten bei einigen spezifischen Tumoren entsprechende prädiktive Korrelationen von hohem TMB-Wert und dem verbesserten Ansprechen auf eine Checkpointinhibitortherapie zeigen, beispielweise bei nichtkleinzelligem Bronchialkarzinom (NSCLC) für Pembrolizumab oder Atezolizumab [14, 15] beim Urothelkarzinom für Atezolizumab [16] oder bei kleinzelligem Lungenkrebs (SCLC) für Nivolumab bzw. für Nivolumab in Kombination mit Ipilimumab [17].

## Kombinationstherapien

Das klinische Problem, dass bei einer Monotherapie mit Checkpointinhibitoren häufig nur wenige Patienten ansprechen, versucht man auch durch den Einsatz von Kombinationstherapien zu überwinden. Über Kombinationen von Therapien mit unterschiedlichen Wirkmechanismen möchte man eine additive, im besten Fall sogar synergistische Wirkung erreichen und/oder die Ausbildung von Resistenzen vermeiden.

Bisher zugelassen sind Kombinationen von PD-1/PD-L1-Inhibitoren mit Chemotherapie, mit CTLA-4-Inhibitor und mit Inhibitoren von vaskulären endothelialen Wachstumsfaktoren (VEGF-Inhibitoren; Tab. 1).

### Kombination mit Chemotherapie

Durch eine Chemotherapie erzielt man antiproliferative und zytotoxische Effekte. Zudem wird postuliert, dass die Tumoren z. B. durch die erhöhte Freisetzung von Antigenen empfänglicher für Immuntherapien gemacht werden könnten [18]. Zudem gibt es präklinische Hinweise darauf, dass eine Chemotherapie in manchen Fällen über die Verminderung der Anzahl der immunsuppressiv wirkenden regulatorischen T‑Zellen (Tregs) die Immunantwort erhöhen könnte [19].

Tatsächlich lässt sich die Überlegenheit einer Kombination von Checkpointinhibitoren mit einer Chemotherapie in der Erstlinientherapie metastasierter Lungenkarzinome nachweisen (Tab. 1). Beispielsweise fand sich für Patienten mit nichtplattenepithelialem Lungenkarzinom eine Ansprechrate von 48 % für die Kombinationstherapie im Vergleich zu 19 % für die Chemotherapie (Studie KEYNOTE-189 [20, 21]). Entsprechend zeigten sich auch für plattenepitheliale Tumore Ansprechraten von 58 % vs. 38 % (Studie KEYNOTE-407 [22]). Demgegenüber fand sich für eine Checkpointinhibitormonotherapie eine histologieunabhängige Ansprechrate von 27,3 % (Studie KEYNOTE-042). Die Ansprechrate der Monotherapie wurde dabei vor allem durch das hohe Ansprechen der Patienten mit stark PD-L1-exprimierenden Tumoren getrieben (Objective Response Rate (ORR) bei Tumor Proportion Score (TPS) ≥50 % betrug 39,5 %; [23, 24]).

### Kombination mit CTLA-4

Eine der ersten Strategien war es, eine Kombination aus Anti-CTLA-4- und Anti-PD-1-Behandlung einzusetzen, um simultan 2 komplementäre, die Immunantwort inhibierende Checkpoints auszuschalten. Kombinationen aus Anti-CTLA-4- und Anti-PD-1-Therapien sind bereits im malignen Melanom und Nierenzellkarzinom zugelassen (Tab. 1). Daten für Patienten mit fortgeschrittenem Melanom zeigten Ansprechraten für die Kombination Nivolumab/Ipilimumab von 59 %, für die Nivolumabmonotherapie von 45 % und 19 % für die Ipilimumabmonotherapie. Die entsprechenden Gesamtüberlebensraten nach 3 Jahren betrugen 58 %, 52 % und 34 % für die Kombinationstherapie bzw. für eine Monotherapie mit Nivolumab oder Ipilimumab [20]. Wichtig zu erwähnen ist hierbei, dass die Kombination von Nivolumab mit Ipilimumab nur bei Patienten mit niedriger Tumor-PD-L1-Expression eine Verlängerung des Gesamtüberlebens (OS) im Vergleich zur Nivolumabmonotherapie gezeigt hat.

### Kombination mit VEGF-Inhibitoren

Ebenfalls zugelassen sind Kombinationen mit Inhibitoren des vaskulären endothelialen Wachstumsfaktors (Vascular Endothelial Growth Factor, VEGF), einerseits als Tyrosinkinaseinhibitoren (z. B. Axitinib) oder als monoklonale Antikörper (z. B. Bevacizumab). Auch hier gibt es präklinische Hinweise für eine synergistische Wirkungsweise. Man vermutet, dass die VEGF-Inhibitoren eine immunsuppressive Tumorumgebung so beeinflussen können, dass eine Immunantwort gegen den Tumor ermöglicht wird [25].

Beispielsweise hat die Kombination von Avelumab und Axitinib in einer Phase-III-Studie bei bisher unbehandelten Patienten mit fortgeschrittenem oder metastasiertem Nierenzellkarzinom eine Überlegenheit gegenüber der bisherigen Standardtherapie mit Sunitinib gezeigt (medianes progressionsfreies Überleben 13,3 vs. 8 Monate und Gesamtansprechrate 53 % vs. 27 % für Avelumab plus Axitinib versus Sunitinib; [7]).

## Ausblick: neue Kombinationstherapien

Neben CTLA‑4 und PD-1/PD-L1 sind verschiedene weitere immunologische Checkpoints auf Immunzellen vorhanden, die die Immunantwort abschwächen oder auch verstärken können. In klinischer Entwicklung sind dabei Kombinationen aus PD-1/PD-L1-Inhibitoren mit weiteren, gegen diese anderen Checkpoints gerichteten Antikörpern, die entweder, wie die bereits zugelassenen Checkpoint-Inhibitoren, die hemmenden Checkpoints blockieren (z. B. LAG‑3, TIM-3) oder die Immunabwehr durch aktivierende Checkpoints direkt stimulieren (z. B. 4‑1BB, OX40, CD40, GITR; [26, 27]).

Dabei sind auch Ansätze in klinischer Entwicklung, die gegen inhibitorische Moleküle auf natürlichen Killerzellen (NK-Zellen) gerichtet sind und damit auch auf die Aktivierung des unspezifischen Immunsystems zielen (z. B. gegen TIGIT gerichtete monoklonale Antikörper; [28]).

Eine weitere Kombination aus verschiedenen Immuntherapien ist der Ansatz, Checkpointinhibitoren zusammen mit Tumorvakzinen einzusetzen [29]. Das therapeutische Prinzip ist dabei, tumorspezifische Antigene „als Impfstoff“ zu nutzen und damit eine zusätzliche, gegen den Tumor gerichtete Immunantwort hervorzurufen. Das Ziel, die Immunantwort weiter zu verstärken, verfolgt man auch über die Kombinationen von Checkpointinhibitoren mit anderen Immuntherapeutika, wie bispezifischen Antikörpern (z. B. Blinatumomab) oder CAR-T-Zellen (z. B. Tisagenlecleucel-Kymriah®; Axicabtagen-Ciloleucel-Yescarta®).

In klinischen Studien werden zudem Kombinationen von direkt in den Tumor injizierten onkolytischen Viren mit Checkpointinhibitoren evaluiert. In klinischer Entwicklung ist auch der Einsatz von zielgerichteten Therapien mit Kinaseinhibitoren (wie z. B. BRAF oder MEK) und Checkpointinhibitoren [30]. Neben diesen medikamentösen Ansätzen gibt es vorläufige klinische Daten, die auf einen möglichen Nutzen einer kombinierten Anwendung mit einer vorausgehenden oder simultanen Radiotherapie hinweisen [31].

Darüber hinaus befinden sich zahlreiche weitere Kombinationen mit Therapeutika auf Basis verschiedenster Wirkmechanismen noch in der präklinischen Entwicklung. Dies veranschaulicht, dass der mit der Zulassung der ersten Checkpointinhibitoren begonnene Wandel in der immunologischen Tumortherapie auch zukünftig fortgesetzt werden kann.

## References

[1] Chen DS, Mellman I (2013). Oncology meets immunology: the cancer-immunity cycle. Immunity.

[2] Sharpe AH (2017). Introduction to checkpoint inhibitors and cancer immunotherapy. Immunol Rev.

[3] SmPC Yervoy annex I summary of product characteristics. https://www.ema.europa.eu/en/documents/product-information/yervoy-epar-product-information_en.pdf. Zugegriffen: 21. Apr. 2020

[4] SMPC Keytruda annex I summary of product characteristics. https://www.ema.europa.eu/en/documents/product-information/keytruda-epar-product-information_en.pdf. Zugegriffen: 17. Mai 2020

[5] SMPC Opdivo annex I summary of product characteristics. https://www.ema.europa.eu/en/documents/product-information/opdivo-epar-product-information_en.pdf. Zugegriffen: 17. Mai 2020

[6] SmPC Tecentriq annex I summary of product characteristics. https://www.ema.europa.eu/en/documents/product-information/tecentriq-epar-product-information_en.pdf. Zugegriffen: 21. Apr. 2020

[7] SMPC Bavencio annex I summary of product characteristics. https://www.ema.europa.eu/en/documents/product-information/bavencio-epar-product-information_en.pdf. Zugegriffen: 17. Mai 2020

[8] SMPC Imfinzi annex I summary of product characteristics. https://www.ema.europa.eu/en/documents/product-information/imfinzi-epar-product-information_en.pdf. Zugegriffen: 17. Mai 2020

[9] SMPC Libtayo annex I summary of product characteristics. https://www.ema.europa.eu/en/documents/product-information/libtayo-epar-product-information_en.pdf. Zugegriffen: 17. Mai 2020

[10] Haanen JBAG, Carbonnel F, Robert C (2017). Management of toxicities from immunotherapy: ESMO Clinical Practice Guidelines for diagnosis, treatment and follow-up. Ann Oncol.

[11] Brahmer JR, Lacchetti C, Schneider BJ (2018). Management of immune-related adverse events in patients treated with immune checkpoint inhibitor therapy: American Society of Clinical Oncology clinical practice guideline. J Clin Oncol.

[12] Cohen EEW, Soulières D, Le Tourneau C (2019). Pembrolizumab versus methotrexate, docetaxel, or cetuximab for recurrent or metastatic head-and-neck squamous cell carcinoma (KEYNOTE-040): a randomised, open-label, phase 3 study. Lancet.

[13] Epar Keytruda (2020) Assessment report Keytruda. EMA/CHMP/591139/2019/corr. https://www.ema.europa.eu/en/documents/variation-report/keytruda-h-c-3820-ii-0065-epar-assessment-report-variation_en.pdf. Zugegriffen: 17. Mai 2020

[14] Rizvi NA, Hellmann MD, Snyder A (2015). Cancer immunology. Mutational landscape determines sensitivity to PD-1 blockade in non-small cell lung cancer. Science.

[15] Gandara DR, Paul SM, Kowanetz M (2018). Blood-based tumor mutational burden as a predictor of clinical benefit in non-small-cell lung cancer patients treated with atezolizumab. Nat Med.

[16] Rosenberg JE, Hoffman-Censits J, Powles T (2016). Atezolizumab in patients with locally advanced and metastatic urothelial carcinoma who have progressed following treatment with platinum-based chemotherapy: a single-arm, multicentre, phase 2 trial. Lancet.

[17] Hellmann MD, Paz-Ares L, Bernabe Caro R (2019). Nivolumab plus Ipilimumab in advanced non-small-cell lung cancer. N Engl J Med.

[18] Zitvogel L, Kepp O, Kroemer G (2011). Immune parameters affecting the efficacy of chemotherapeutic regimens. Nat Rev Clin Oncol.

[19] Włodarczyk M, Ograczyk E, Kowalewicz-Kulbat M, Druszczyńska M, Rudnicka W, Fol M (2018). Effect of cyclophosphamide treatment on central and effector memory T cells in mice. Int J Toxicol.

[20] Wolchok JD, Chiarion-Sileni V, Gonzalez R (2017). Overall survival with combined nivolumab and ipilimumab in advanced melanoma. N Engl J Med.

[21] Gandhi L, Rodríguez-Abreu D, Gadgeel S (2018). Pembrolizumab plus chemotherapy in metastatic non-small-cell lung cancer. N Engl J Med.

[22] Paz-Ares L, Luft A, Vicente D (2018). Pembrolizumab plus chemotherapy for squamous non-small-cell lung cancer. N Engl J Med.

[23] Mok TSK, Wu Y-L, Kudaba I (2019). Pembrolizumab versus chemotherapy for previously untreated, PD-L1-expressing, locally advanced or metastatic non-small-cell lung cancer (KEYNOTE-042): a randomised, open-label, controlled, phase 3 trial. Lancet.

[24] Reck M, Rodríguez-Abreu D, Robinson AG (2016). Pembrolizumab versus chemotherapy for PD-L1-positive non-small-cell lung cancer. N Engl J Med.

[25] Fukumura D, Kloepper J, Amoozgar Z, Duda DG, Jain RK (2018). Enhancing cancer immunotherapy using antiangiogenics: opportunities and challenges. Nat Rev Clin Oncol.

[26] Marin-Acevedo JA, Dholaria B, Soyano AE, Knutson KL, Chumsri S, Lou Y (2018). Next generation of immune checkpoint therapy in cancer: new developments and challenges. J Hematol Oncol.

[27] Mahoney KM, Rennert PD, Freeman GJ (2015). Combination cancer immunotherapy and new immunomodulatory targets. Nat Rev Drug Discov.

[28] Manieri NA, Chiang EY, Grogan JL (2017). TIGIT: a key inhibitor of the cancer immunity cycle. Trends Immunol.

[29] Ott PA, Hu Z, Keskin DB (2017). An immunogenic personal neoantigen vaccine for patients with melanoma. Nature.

[30] Cyprian FS, Akhtar S, Gatalica Z, Vranic S (2019). Targeted immunotherapy with a checkpoint inhibitor in combination with chemotherapy: a new clinical paradigm in the treatment of triple-negative breast cancer. Bosn J Basic Med Sci.

[31] Ko EC, Formenti SC (2018). Radiotherapy and checkpoint inhibitors: a winning new combination?. Ther Adv Med Oncol.

